# Synergistic Grain Boundary and Phonon Engineering Enables Ultrahigh Thermoelectric Performance in Mg_3_(Sb, Bi)_2_‐Based Materials

**DOI:** 10.1002/advs.77449

**Published:** 2026-08-30

**Authors:** Jingdan Lei, Kai Xu, Kunpeng Zhao, Junzhuo Zhou, Haotian Gao, Yifan Yuan, Tian‐Ran Wei, Min Zhu, Qihao Zhang, Franck Gascoin, Xun Shi

**Affiliations:** ^1^ Anyang Key Laboratory of Optoelectronic Information Materials School of Physics and Electrical Engineering Anyang Normal University Anyang China; ^2^ State Key Laboratory of Metal Matrix Composites School of Materials Science and Engineering Shanghai Jiao Tong University Shanghai China; ^3^ Institute of Information Functional Materials Shanghai Key Laboratory of Atomic‐Level Intelligent Manufacturing of Materials and Devices School of Materials Science and Engineering Shanghai Jiao Tong University Shanghai China; ^4^ State Key Laboratory of Advanced Fiber Materials College of Materials Science and Engineering Donghua University Shanghai China; ^5^ School of Integrated Circuits Shanghai Jiao Tong University Shanghai China; ^6^ Laboratoire CRISMAT ENSICAEN UNICAEN CNRS Normandie Université (UMR 6508) Caen France; ^7^ State Key Laboratory of High Performance Ceramics Shanghai Institute of Ceramics Chinese Academy of Sciences Shanghai China

**Keywords:** carrier mobility, electrical transport, grain boundary scattering, Mg_3_(Sb, Bi)_2_, thermoelectric

## Abstract

Thermoelectric Mg_3_(Sb, Bi)_2_ has emerged as a rising star in virtue of its promising thermoelectric performance and abundant constituent elements. However, its practical application is hindered by challenges in synthesis and the strong coupling between electrical and thermal transport properties. Herein, we propose a dual‐pronged strategy that combines grain boundary engineering and phonon engineering to decouple electron and phonon transport in Mg_3_(Sb, Bi)_2_. By employing a tantalum‐tube encapsulated melting technique together with Cu doping, the grain size is markedly increased to ∼25 µm, effectively suppressing carrier grain boundary scattering and enabling single‐crystal‐like electrical transport with a high carrier mobility exceeding 200 cm^2^ V^−1^ s^−1^. Meanwhile, atomic‐scale alloy disorder and nanoscale Mg_2_Cu precipitates introduce strong phonon scattering, leading to an ultralow lattice thermal conductivity of 0.36 W m^−1^ K^−1^. Eventually, a peak *zT* of 1.9 at 750 K and a high conversion efficiency of 10.6% at *ΔT* = 488 K are achieved, demonstrating the competitive performance of these materials and devices. This study not only provides new insights into the manipulation of grain boundary scattering, but also demonstrates the great promise of Mg_3_(Sb, Bi)_2_ for waste‐heat harvesting.

## Graphical abstract

Ta encapsulated melting and Cu doping synergistically engineer grain boundaries and phonon scattering in Mg_3_(Sb,Bi)_2_, enabling single‐crystal‐like carrier transport and high thermoelectric performance.

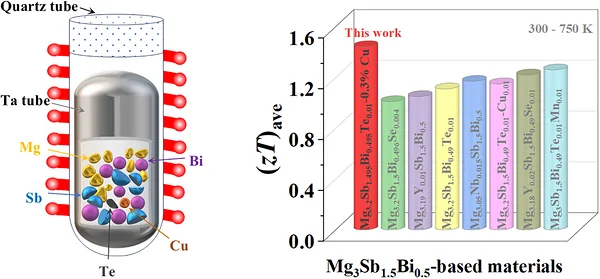

## 1 Introduction

Waste heat minimization and recovery represent an effective strategy to alleviate the global energy crisis and environmental pollution. Solid‐state thermoelectric (TE) technology, which enables the mutual conversion of waste heat into electricity, stands out as a highly reliable approach for power generation and solid‐state refrigeration [1, 2, 3]. The energy conversion efficiency of TE materials is mainly gauged by the dimensionless figure of merit, *zT* = $\frac{S^{2}\sigma }{\kappa }T$, where *S*, *σ*, *κ*, and *T* are the Seebeck coefficient, electrical conductivity, thermal conductivity, and absolute temperature, respectively [4, 5]. The term *S* ^2^ *σ*, known as power factor (*PF*), is a critical indicator that represents the electrical properties. A superior TE material should have a high *PF* for retaining high output power and a large *zT* for maximizing the conversion efficiency.

Large‐scale deployment of TEs demands inexpensive, eco‐friendly, and nontoxic materials with high TE performance. State‐of‐the‐art TE materials such as Bi_2_Te_3_ and PbTe rely on scarce or toxic elements, limiting their widespread application. Recently, Mg_3_(Sb, Bi)_2_‐based materials stand out as they not only exhibit high TE performance and excellent mechanical properties, but also are rich in reserve of constituent elements [6, 7, 8, 9, 10, 11]. Mg_3_(Bi, Sb)_2_ adopts a hexagonal structure, featuring intrinsic large lattice anharmonicity [12]. Its electronic structure, characterized by high valley degeneracy and low effective mass, enables excellent carrier transport [7]. Compositional tuning of the Sb/Bi ratio further allows simultaneous optimization of electronic and phononic properties [13]. Following the pioneering work by Tamaki et al. [6] and Iversen et al. [7], substantial efforts have been devoted to enhancing the TE performance of Mg_3_(Sb, Bi)_2_ via chemical doping [14, 15, 16, 17, 18], band structure engineering [19, 20], microstructure modulation [21, 22, 23, 24, 25], and carrier scattering manipulation [26, 27, 28, 29, 30]. The TE figure of merit *zT* at high temperature has been boosted to 1.7–2.0.

Despite these advances, the development of high‐performance Mg_3_(Sb, Bi)_2_‐based materials faces challenges in material synthesis and TE transport regulation. The synthesis of phase‐pure Mg_3_(Sb, Bi)_2_ is complicated by the high chemical activity and volatility of Mg. Various synthesis techniques have been explored, including multi‐step solid‐state reactions [31], self‐propagating high‐temperature synthesis [32], induction sintering [33], and ball milling [6]. The multi‐step approach suffers from prolonged processing times and poor compositional homogeneity, while self‐propagating synthesis exhibits limited reaction controllability and product instability. Ball milling is a more accessible approach, yet it commonly introduces excessive grain refinement and oxidation, which negatively affect transport properties. In terms of electrical transport, grain boundary (GB) scattering has been identified as a major factor limiting carrier mobility, especially near room temperature [26]. Enlarging grain size through techniques such as prolonged annealing [28], high‐temperature sintering [29], or liquid‐phase sintering [27] can effectively suppress GB scattering and enhance carrier mobility. However, these approaches risk substantial Mg loss and lead to compositional deviation. Regarding thermal transport, alloying and doping strategies have been applied to reduce the lattice thermal conductivity by introducing phonon scattering centers [17, 19, 34]. Nonetheless, these modifications often lead to concurrent carrier scattering. Nanocomposite engineering, another popular approach to reduce lattice thermal conductivity, faces obstacles in achieving uniform dispersion of secondary phases [24]. Therefore, developing a robust methodology to decouple electron and phonon transport while ensuring single‐phase Mg_3_(Sb, Bi)_2_ synthesis is imperative.

In this work, we address this challenge by implementing a coupled microstructural and phononic design strategy. A tantalum‐tube encapsulated melting technique enables robust synthesis of coarse‐grained materials, while Cu addition modifies grain boundaries and introduces Mg_2_Cu nanoprecipitates. This dual approach simultaneously enhances carrier mobility and suppresses lattice thermal conductivity, leading to single‐crystal‐like transport behavior in a polycrystalline system. As a result, a high peak *zT* of 1.9 at 750 K is achieved, together with a module‐level conversion efficiency of 10.6% under a *ΔT* of 488 K, demonstrating the effectiveness of this strategy for both materials optimization and practical energy conversion applications.

## 2 Results and Discussion

**FIGURE 1:**
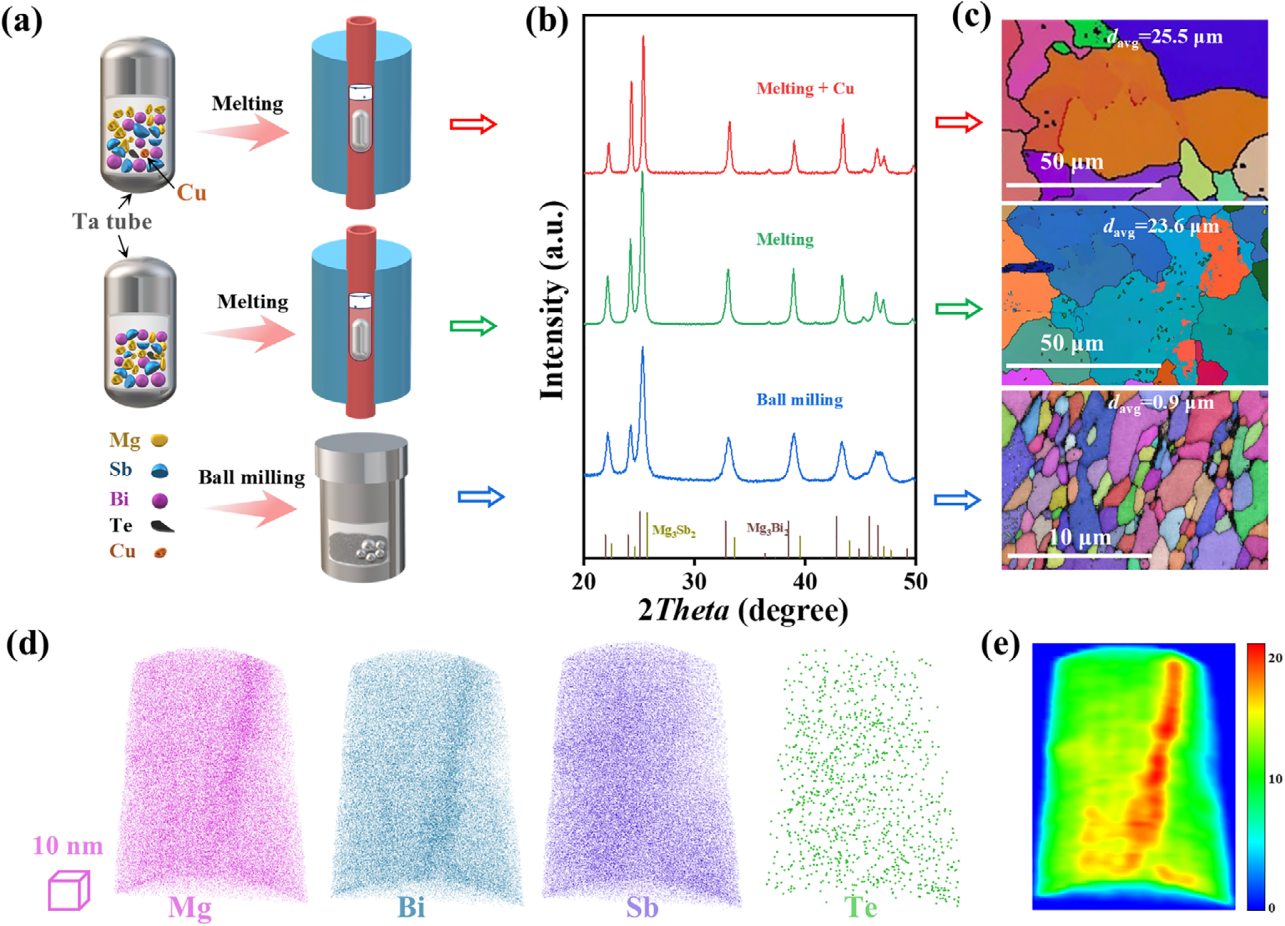
Crystal structure and microstructure for Mg_3.2_Bi_1.195_Sb_0.795_Te_0.01_. (a) Schematic diagram of the two different methods for synthesizing Mg_3.2_Bi_1.195_Sb_0.795_Te_0.01_:ball milling and melting, as well as melted Mg_3.2_Bi_1.195_Sb_0.795_Te_0.01_‐0.3% Cu. (b) Powder XRD patterns at room temperature and (c) corresponding grain size revealed by electron back‐scattering diffraction (EBSD) measurements. (d) 3D elemental distribution of Mg, Bi, Sb, and Te obtained from atom probe tomography (APT) and (e) corresponding 2D atomic density contour mapping. The grain boundary obviously has a higher atomic density and is enriched in Mg and Bi.

To compare the effects of different synthesis methods on the properties, we synthesized two Mg_3.2_Bi_1.195_Sb_0.795_Te_0.01_ samples with identical compositions using both ball milling and tantalum‐tube encapsulated melting techniques. In addition, to clarify the role of Cu doping, we prepared an additional Cu‐doped Mg_3.2_Bi_1.195_Sb_0.795_Te_0.01_ sample by the tantalum‐tube encapsulated melting technique, namely the Mg_3.2_Bi_1.195_Sb_0.795_Te_0.01_‐0.3% Cu sample. Tantalum was selected as the encapsulation material because of its high melting point, low chemical reactivity, and good compatibility with Mg, Sb, and Bi under the present processing conditions [35]. More importantly, the small, fully sealed space effectively suppresses Mg evaporation and maintains a Mg‐rich environment throughout melting and solidification. All samples exhibit diffraction peaks consistent with the trigonal Mg_3_Sb_2_ structure, with no detectable secondary phases (Figure 1b). The reduced full width at half maximum (FWHM) in melted samples (Table S1) indicates improved crystallinity and enlarged grain size compared to ball‐milled counterparts. Scanning electron microscopy (SEM) images reveal homogeneous elemental distributions in both ball‐milled and melted Cu‐free samples, whereas Mg‐Cu precipitate enrichment is observed in the Cu‐doped sample (Figure S1). Moreover, no Ta signal or Ta‐containing secondary phase was detected, indicating negligible Ta‐related contamination or reaction under the present processing conditions.

The grain size and morphology were evaluated through the electron backscattering diffraction (EBSD) method. As shown in Figure 1c, the average grain size of the ball‐milled sample is 0.9 µm, consistent with the values (0.2–3 µm) reported in the literature [10, 36, 37]. In contrast, the melted sample exhibits a much larger grain size of 23.6 µm, one to two orders of magnitude greater than that of the ball‐milled sample. The addition of Cu has a slight effect on the grain growth, with the mean grain size further increasing to 25.5 µm. Previous studies have shown that Cu can combine with Mg to form Mg_2_Cu, which acts as a liquid‐phase sintering aid and promotes grain coarsening during sintering [27]. For samples with initially fine grains, Cu can significantly increase the grain size from a few micrometers to several tens of micrometers. However, for our melted sample, which already has relatively large grains, the observable effect of Cu is less pronounced.

We further performed 3D atom probe tomography (APT) analysis to determine the local chemical compositions and element distribution of the Cu‐doped sample. APT is a cutting‐edge technique that enables near‐atomic scale elemental mapping with a chemical sensitivity down to tens of parts per million (ppm) [38]. As shown in Figure 1d, Mg, Bi, Sb, and Te are clearly detected, whereas no trace of Cu is observed in the analyzed volume. Because APT probes only an extremely small, site‐specific region, the selected specimen may not contain the sparsely distributed Cu‐rich precipitates. Nevertheless, the high chemical sensitivity of APT indicates that the Cu content within the Mg_3_(Sb, Bi)_2_ lattice is extremely low. A stripe‐like region enriched in Mg and Bi but depleted in Sb is also observed in the APT reconstruction. Away from this region, all elements, including dilute Te, are uniformly distributed. The corresponding 2D atomic density contour mapping reveal a higher atomic density within the stripe and different densities on its two sides, consistent with distinct crystallographic orientations [39] and confirming that the stripe represents a grain boundary. Notably, the Mg‐rich grain boundary observed here contrasts with the Mg‐deficient boundaries reported previously [25, 39]. Compensating for the Mg deficiency is essential to minimize the unoccupied trap state density and mitigate the depletion of electrons, leading to the reduction of the potential barrier. Therefore, the richness of Mg along the boundaries in our work is expected to give a high carrier mobility and superior electrical transport.

**FIGURE 2:**
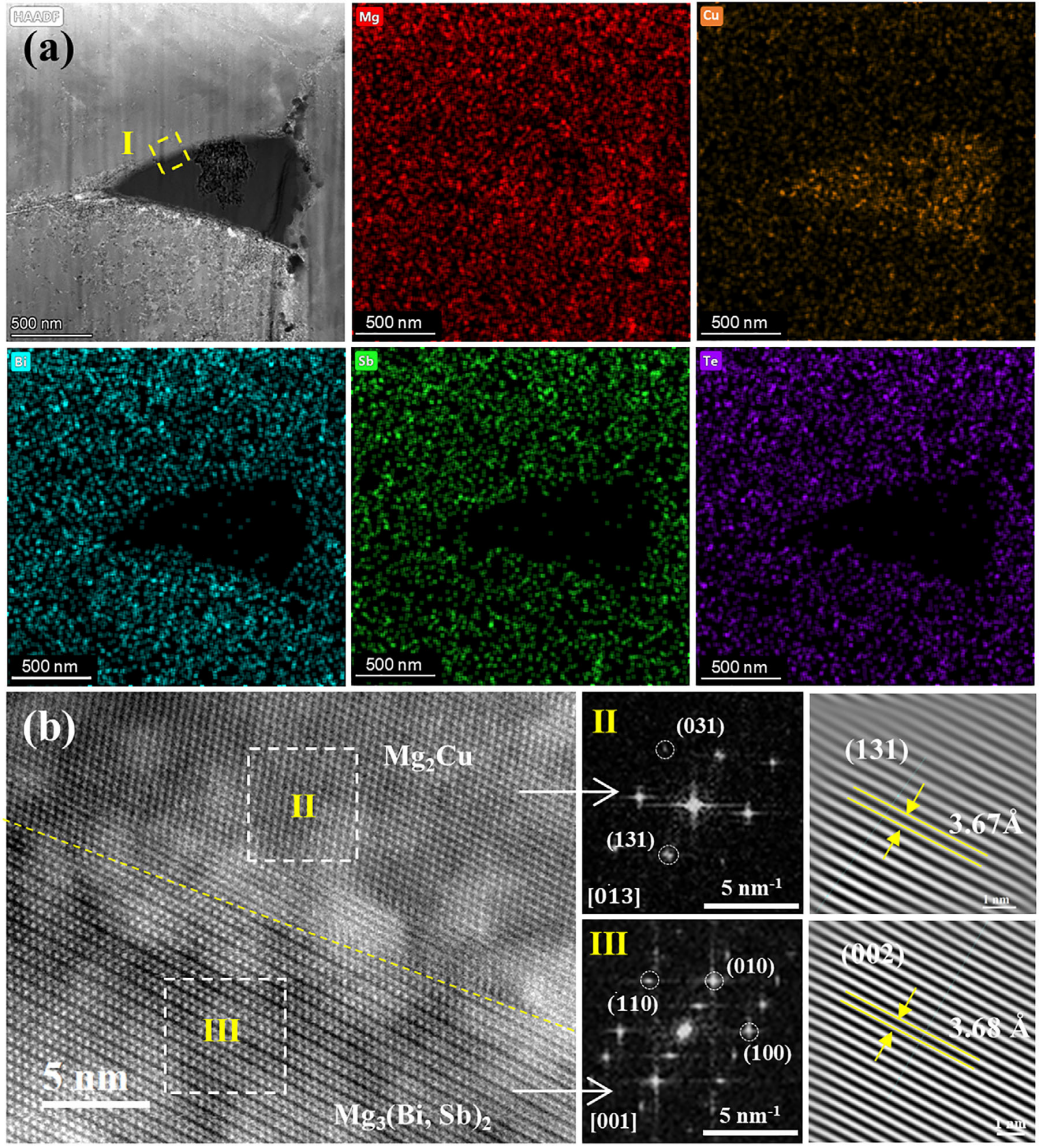
Morphology of the grain boundaries for Mg_3.2_Bi_1.195_Sb_0.795_Te_0.01_‐0.3% Cu. (a) High‐angle annular dark‐field scanning transmission electron microscopy (HAADF‐STEM) image and the corresponding EDS mapping. (b) Atomic‐resolution HAADF‐STEM images of region I in Figure 2a. The FFT patterns and IFFT analysis of region II grain boundary and region III grain phases, conforming a coherent interface between the matrix and Mg_2_Cu.

To further investigate the chemical state of Cu in the Cu‐doped sample, we conducted aberration‐corrected scanning transmission electron microscopy (AC‐STEM) analysis. As shown in Figure 2a, the high‐angle annular dark‐field (HAADF) image and corresponding energy‐dispersive X‐ray spectroscopy (EDS) mapping indicate that Cu is located at the triple junction of the Mg_3_(Sb, Bi)_2_ grain boundaries. Furthermore, Cu does not exist in its metallic form but rather forms an alloy with Mg. Figure 2b presents a high‐resolution HAADF image of the two‐phase interface at a local scale. It is evident that the Cu‐rich region corresponds to Mg_2_Cu, and a coherent interface is formed between Mg_2_Cu and Mg_3_(Sb, Bi)_2_. This interface coherence is confirmed by the similarity in the fast Fourier transform (FFT) patterns of both phases and the comparable interplanar spacings observed through inverse FFT (IFFT) analysis. The complementary APT and STEM‐EDS analyses confirm that Cu is scarcely incorporated into the Mg_3_(Sb, Bi)_2_ lattice but is heterogeneously distributed at grain boundaries. Our previous studies have demonstrated that Mg_2_Cu can serve as a liquid‐phase sintering aid, promoting grain growth during the high‐temperature (893 K) and high‐pressure (50 MPa) sintering process [27]. Additionally, these Mg_2_Cu nanoprecipitates could effectively scatter the phonons and thereby suppress the lattice thermal conductivity.

**FIGURE 3:**
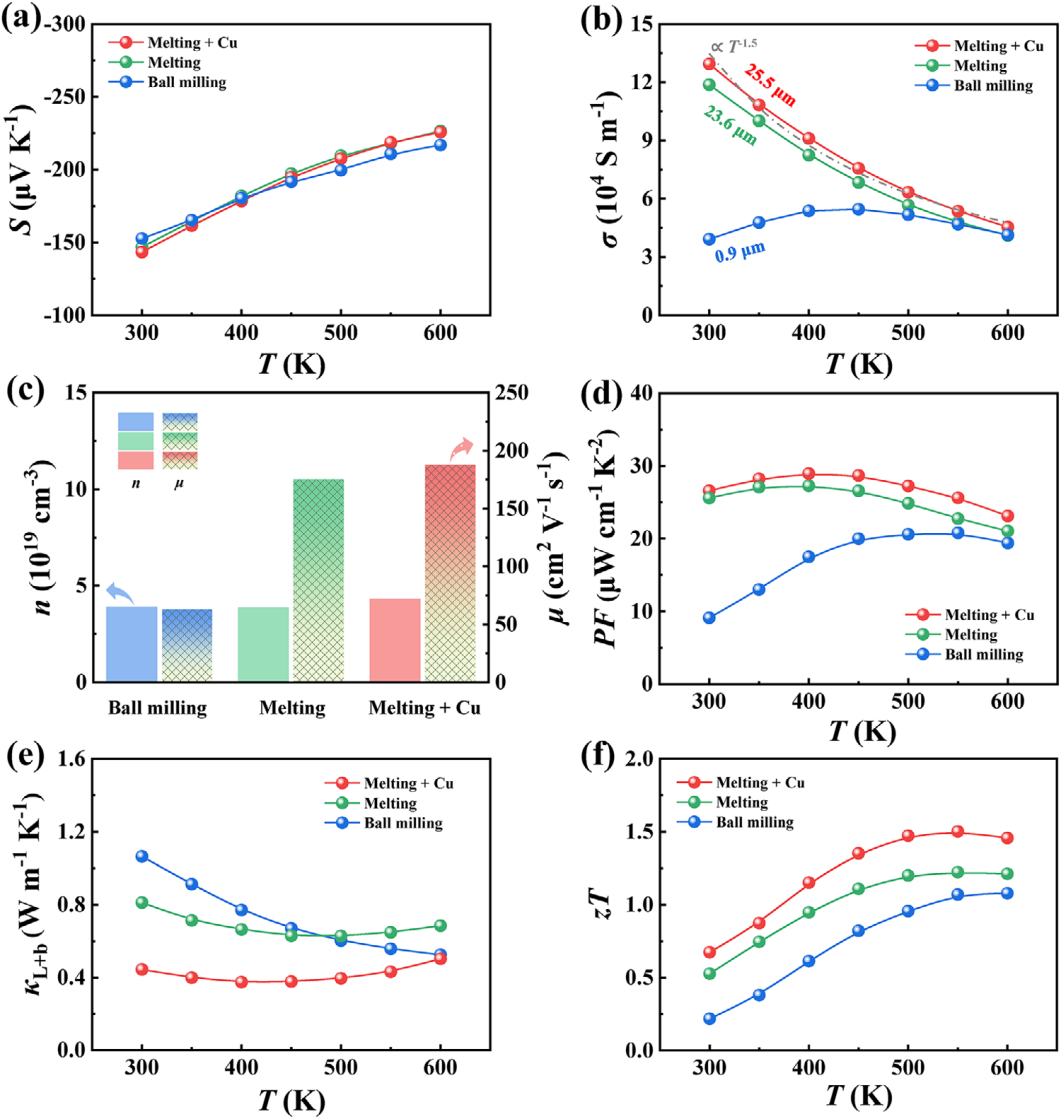
TE properties for Mg_3.2_Bi_1.195_Sb_0.795_Te_0.01_ prepared by ball milling, Mg_3.2_Bi_1.195_Sb_0.795_Te_0.01_ and Mg_3.2_Bi_1.195_Sb_0.795_Te_0.01_‐0.3% Cu prepared by melting. Temperature dependent (a) Seebeck coefficient *S*, (b) electrical conductivity *σ*, (d) power factor *PF*, (e) lattice and bipolar thermal conductivity *κ*_L+b_, and (f) TE figure of merit *zT*. (c) Room temperature Hall carrier concentration *n* and Hall mobility *µ*.

The enlarged grain size and modified grain boundaries have a large positive impact on the electrical transports of Mg_3_(Sb, Bi)_2_‐based samples. Although the Seebeck coefficients of the three samples are nearly identical (Figure 3a), the temperature dependent electrical conductivity *σ* of the melted samples is qualitatively different from that of the fine‐grained ball milled sample at low temperatures (Figure 3b). This suggests that the carrier concentration is essentially the same across all samples and that it is primarily the carrier mobility that is affected. Attributed to the strong grain boundary scattering of electrons, the *σ* of ball‐milled sample increases with temperature around room temperature. Conversely, the *σ* of melted samples shows a *T* ^−1.5^ temperature dependence that resembles the single‐crystal conductivity behavior. This indicates that the charge carriers are predominantly scattered by phonons while the grain boundary scattering effect has been largely suppressed for the melted samples. Consistent with this interpretation, the measured carrier concentration *n* remains nearly identical across samples prepared by different synthesis methods, whereas the carrier mobility *µ* shows significant variation (Figure 3c). The mobility *µ* of the melted Cu‐free sample reaches 175 cm^2^ V^−1^ s^−1^, which is three times higher than that of the ball‐milled sample. The Cu‐doped sample achieves an even higher mobility of 188 cm^2^ V^−1^ s^−1^ due to the enlarged grain size and modified GBs. Owing to the enhanced mobility, the power factor *PF*s of the melted samples are significantly higher than that of the ball‐milled sample, particularly near room temperature (Figure 3d). The melted Cu‐free and Cu‐doped samples achieve room‐temperature *PF*s of 25.2 and 26.6 µW cm^−1^ K^−2^, respectively, marking a 150% and 170% improvement over the ball‐milled sample.

The synthesis method and Cu doping also exert a significant influence on the thermal transport. Figure S2 shows the total thermal conductivity *κ* as a function of temperature. At room temperature, the melted Cu‐free sample exhibits the highest thermal conductivity (1.43 W m^−1^ K^−1^), while the melted Cu‐doped sample shows the lowest (1.12 W m^−1^ K^−1^). After subtracting the electronic contribution, the combined lattice and bipolar thermal conductivity (*κ*_L+b_) was obtained (Figure 3e). At high temperatures, the melted samples show a notable bipolar thermal conductivity, causing an upward deviation of *κ*_L+b_. At room temperature, the bipolar contribution is negligible. Despite its larger grains, the melted sample exhibits a lower *κ*_L_ than the ball‐milled sample. This behavior is mainly attributed to the more homogeneous atomic‐scale distribution of Sb and Bi achieved by tantalum‐tube encapsulated melting, which enhances alloy‐disorder scattering. Meanwhile, the sealed environment suppresses Mg oxidation and consequently inhibits the formation of high‐thermal‐conductivity MgO [40]. These effects outweigh the reduced grain‐boundary scattering associated with the larger grains, resulting in a lower *κ*_L_ for the melted sample. Notably, across the same synthesis method, the Cu‐doped sample shows a much lower *κ*_L_ than the Cu‐free counterpart over the entire temperature range. Specifically, the room‐temperature *κ*_L_ of the melted Cu‐doped sample is only 0.44 W m^−1^ K^−1^, nearly half that of the Cu‐free sample. This suppressed phonon transport is attributed not only to the intense phonon scattering at the interfaces of Mg_2_Cu nanoprecipitates but also to the subtle shifts in matrix stoichiometry. Since the added Cu reacts with the excess Mg to form the Mg_2_Cu phase, the effective Mg‐content within the Mg_3_(Sb, Bi)_2_ lattice is altered. This variation in the actual cation/anion ratio can further perturb the lattice periodicity and enhance point‐defect scattering, a synergistic effect that contributes to the extremely low *κ*_L_ observed here. As a result of the enhanced *µ* and reduced *κ*_L_, a peak *zT* value of 1.5 is finally achieved at 550 K for the melted Cu‐doped sample (Figure 3f). This value surpasses that of most previously reported Mg_3_(Sb, Bi)_2_‐based materials within the same temperature range.

**FIGURE 4:**
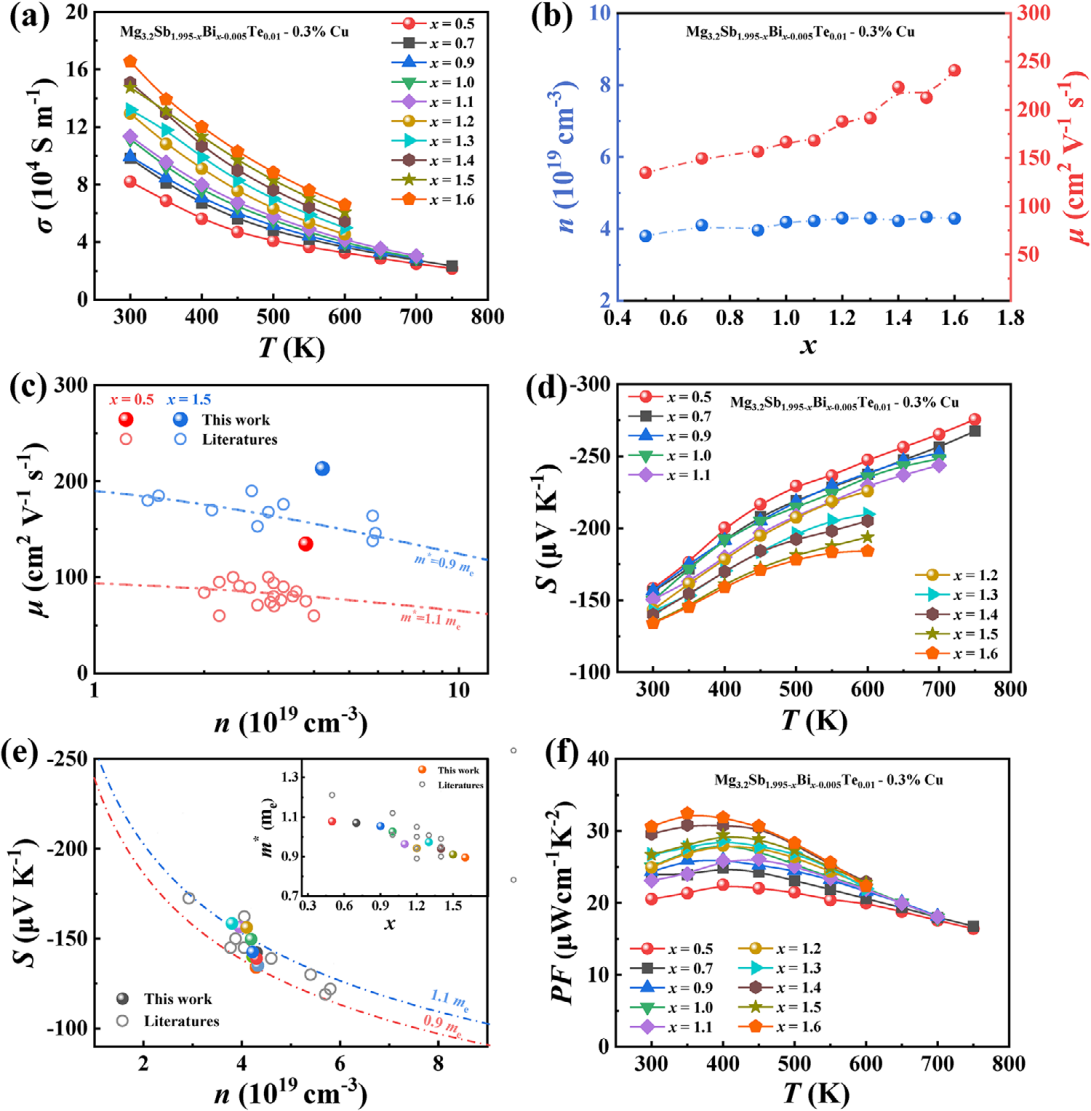
Electrical transport properties for Mg_3.2_Sb_1.995‐_ *_x_* Bi*_x‐_*_0.005_Te_0.01_‐0.3% Cu (0.5 ≤ *x* ≤ 1.6) prepared by the tantalum‐tube encapsulated melting method. (a) Temperature dependent electrical conductivity *σ*, (d) Seebeck coefficient *S*, and (f) power factor *PF*. (b) Room temperature Hall carrier concentration *n* and Hall mobility *µ* as a function of Bi content *x*. (c) Hall carrier concentration dependent Hall mobility for Mg_3.2_Sb_1.995‐_ *_x_* Bi*_x‐_*_0.005_Te_0.01_‐0.3% Cu (*x* = 0.5, 1.5) with a comparison to reported data. The dashed lines are obtained using effective mass *m* ^*^ are 0.9 *m*_e_ and 1.1 *m*_e_ with a deformation potential coefficient *E*_def_ of 20 eV. The open circles represent the data of Mg_3_(Bi, Sb)_2_ with the same Bi content from literatures [4, 8, 9, 10, 18, 20, 28, 36, 40, 41, 42, 43, 44, 45, 46, 47, 48]. (e) Room temperature Seebeck coefficient *S* as a function of carrier concentration *n*. The dashed lines denote the calculated Pisarenko plots with effective mass *m* ^*^ of 0.9 *m*_e_ and 1.1 *m*_e_. The inset shows the effective mass *m* ^*^ as a function of Bi content *x* [9, 40, 49, 51, 52].

Tuning the Sb/Bi ratio is an effective strategy for matching the TE performance to different operating‐temperature windows [19, 20, 40]. Increasing the Bi content enhances carrier mobility, whereas increasing the Sb content widens the band gap and suppresses bipolar excitation. Meanwhile, Sb/Bi mixing introduces strong alloy‐disorder scattering of phonons. Therefore, to further optimize the TE performance at high temperatures, we synthesized a series of Cu‐doped Mg_3.2_Sb_1.995‐_ *_x_* Bi*_x‐_*_0.005_Te_0.01_‐0.3% Cu samples with varying Sb/Bi ratios using the tantalum‐tube encapsulated melting method. All samples were confirmed to be phase‐pure (Figure S3a). The refined lattice parameters increase linearly with increasing Bi content, following Vegard's law (Figures S3b and S4). The electrical conductivity (*σ*) of all samples shows a negative temperature dependence, indicating minimal carrier scattering by grain boundaries. With increasing Bi content, *σ* gradually increases due to the nearly unchanged carrier concentration *n* and enhanced carrier mobility *µ* (Figure 4b). Notably, for samples with the same Sb/Bi ratio, our samples demonstrate significantly higher *µ* compared to previously reported Mg_3_(Sb,Bi)_2_ samples [4, 8, 9, 10, 18, 20, 28, 36, 40, 41, 42, 43, 44, 45, 46, 47, 48] (Figure 4c). Opposite to the trend observed in *σ*, the Seebeck coefficient (*S*) increases progressively as the Bi content decreases (Figure 4d). Specifically, the room temperature *S* increase from −134 µV K^−1^ for *x* = 1.6 sample to −158 µV K^−1^ for *x* = 0.5 sample. The increment is getting more pronounced at high temperature because the bipolar effect is suppressed in Sb‐rich samples. The experimental *S* vs. *n* data (symbols) together with the theoretical Pisarenko plot (dashed lines) are shown in Figure 4e. The derived effective mass *m* ^*^ decreases from 1.1 *m*_e_ for *x* = 0.5 sample to 0.9 *m_e_* for *x* = 1.6. Previous first‐principles calculations [49, 50] show that the less spatially confined Bi 6*p* orbitals interact more strongly with the Mg 3*s* orbitals than the Sb 5*p* orbitals, producing a more dispersive conduction band and reducing both the band and inertial effective masses. Because the Seebeck coefficient is positively correlated with the density‐of‐states effective mass, whereas carrier mobility is inversely related to the inertial effective mass, this band evolution explains the simultaneous decrease in *S* and increase in *µ* with increasing Bi content. Similar trends have been reported previously [20, 22, 40]. Figure 4f plots the temperature dependent power factor *PF*. A maximum *PF* of 32.5 µW cm^−1^ K^−2^ is achieved at 350 K for the *x* = 1.6 sample, which is 50% higher than that of the *x* = 0.5 sample and even surpassed many textured n‐type Bi_2_(Te, Se)_3_ materials.

**FIGURE 5:**
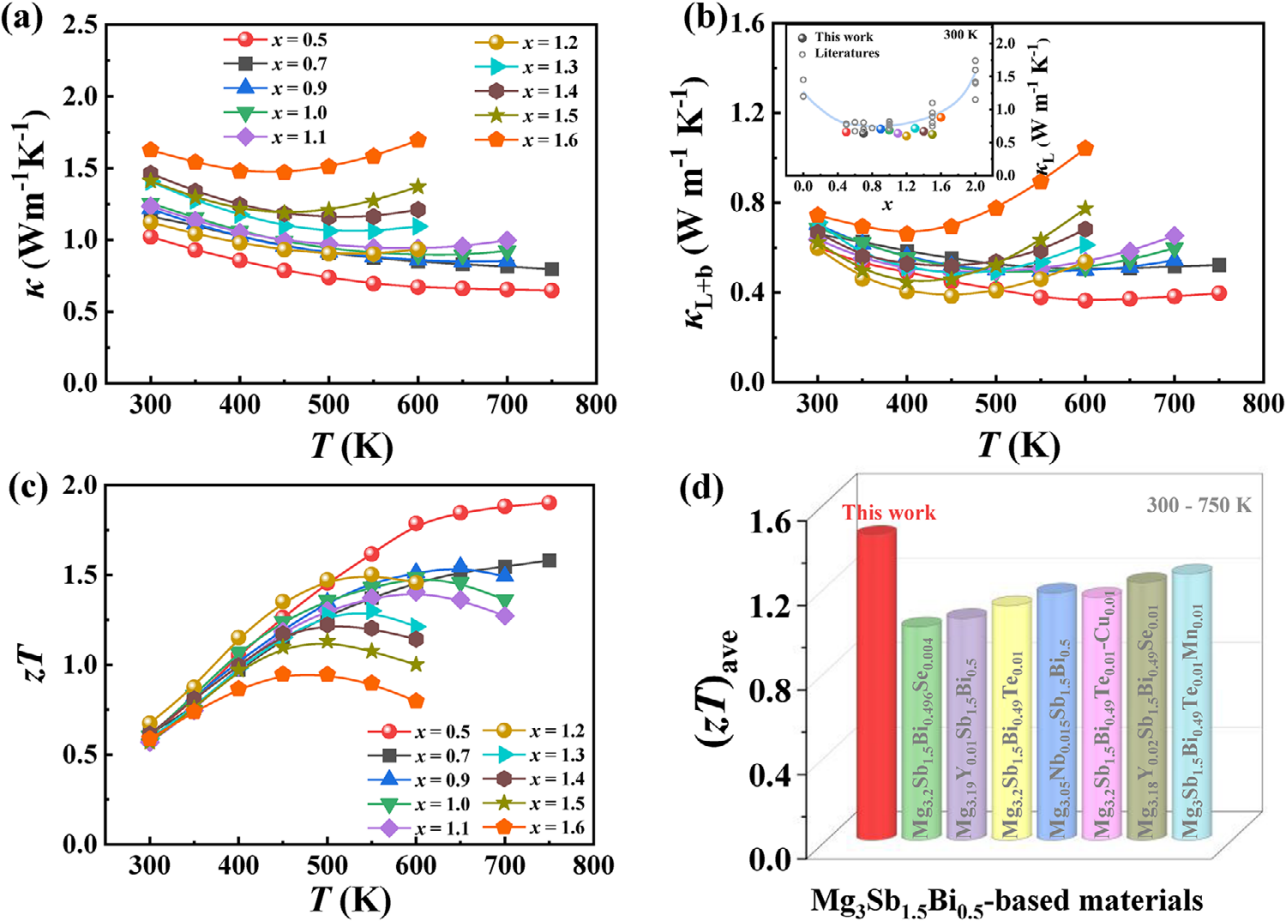
Thermal transport properties and TE figure of merit *zT* for Mg_3.2_Sb_1.995‐_ *_x_* Bi*_x‐_*_0.005_Te_0.01_‐0.3% Cu (0.5 ≤ *x* ≤ 1.6) prepared by tantalum‐tube encapsulated melting method. Temperature dependence of (a) total thermal conductivity *κ*, (b) lattice and bipolar thermal conductivity *κ*_L+b_, and (c) *zT* values. The *x =* 0.5 and *x =* 1.2 samples exhibit exceptional low‐to‐medium temperature TE performance. The inset in Figure 5b shows the room temperature *κ*_L_ as a function of Bi content [4, 9, 29, 33, 41, 49]. (d) Extraordinary average *zT* (*zT*)_avg_ of Mg_3.2_Bi_0.495_Sb_1.495_Te_0.01_‐0.3% Cu in comparison with the data of Mg_3_Bi_0.5_Sb_1.5_‐based materials from literatures [8, 10, 16, 22, 44, 48, 53].

Figure 5a presents the temperature dependence of total thermal conductivity *κ* for Mg_3.2_Sb_1.995‐_ *_x_* Bi*_x‐_*_0.005_Te_0.01_‐0.3% Cu (0.5 ≤ *x* ≤ 1.6) samples. With increasing Bi content, the thermal conductivity shows an overall increasing trend, primarily due to the enhanced electronic contribution. After subtracting the electronic thermal conductivity using the Wiedemann‐Franz relation, the combined lattice and bipolar contribution *κ*_L+b_ was obtained, as shown in Figure 5b. For the *x* = 1.6 sample, *κ*_L+b_ begins to increase above approximately 450 K, whereas this upturn shifts to higher temperatures as the Bi content decreases. This behavior does not indicate an increase in the intrinsic lattice contribution. Instead, increasing Bi narrows the band gap and enhances bipolar excitation, thereby increasing *κ*_b_. In the Sb‐rich *x* = 0.5 sample, the wider band gap effectively suppresses bipolar transport, allowing *κ*_L+b_ to reach an extremely low value of approximately 0.36 W m^−1^ K^−1^ at 600 K.

At room temperature, the bipolar contribution is negligible, and the measured *κ*_L_ values for 0.5 ≤ x ≤ 1.6 remain within approximately 0.6‐0.7 W m^−1^ K^−1^, substantially lower than those of the reported Mg_3_Sb_2_ and Mg_3_Bi_2_ end members. The composition‐dependent *κ*_L_ exhibits a U‐shaped trend, demonstrating strong phonon scattering by Sb/Bi alloy disorder (see the inset in Figure 5b). To quantitatively evaluate this effect, the Klemens alloy‐scattering model was employed by separating the point‐defect scattering parameter *Γ* into mass‐fluctuation and strain‐field‐fluctuation terms, *Γ* = *Γ*_M_ + *Γ*_S_ (Figure S5). The model reproduces the experimental composition dependence of *κ*_L_ well, while the comparable magnitudes of *Γ*_M_ and *Γ*_S_ indicate that both contributions are important. The relatively small variation in *κ*_L_ within the investigated composition range reflects the limited change in alloy‐disorder scattering after a sufficiently high alloying level is reached. Moreover, the lower *κ*_L_ values of our samples compared with previously reported Mg_3_Sb_2‐_ *_x_* Bi*_x_* materials of similar compositions are attributed to additional phonon scattering by Mg_2_Cu nanoprecipitates and their interfaces.

Owing to the combination of excellent electrical properties and low lattice thermal conductivity, our Mg_3.2_Sb_1.995‐_ *_x_* Bi*_x‐_*_0.005_Te_0.01_‐0.3% Cu samples exhibit outstanding TE performance. As shown in Figure 5c, the temperature corresponding to the peak *zT* values gradually shifts to higher temperatures with increasing Sb content. Notably, the *x* = 0.5 sample achieves a peak *zT* of 1.9 at 750 K and a high average *zT* (*zT*_avg_) of 1.44 over 300–750 K (Figure 5d), demonstrating competitive performance among reported n‐type Mg_3_(Sb,Bi)_2_‐based materials. Figure S6 presents a comparison of the TE properties between Cu‐containing and Cu‐free samples, highlighting the beneficial effects of Cu addition on regulating electron‐phonon transport and enhancing TE performance. The thermal stability was evaluated through repeated TE measurements (Figure S7). Four measurements were conducted for the *x* = 0.5 sample up to 773 K, while the *x* = 1.2 sample underwent three heating measurements and two heating‐cooling cycles between 300 and 600 K. The variations in *S*, *σ*, and *κ* remained within ±5%, ±4%, and ±10%, respectively. Post‐cycling EDS analysis further revealed no obvious compositional changes or elemental segregation (Figure S8), confirming good thermal stability.

**FIGURE 6:**
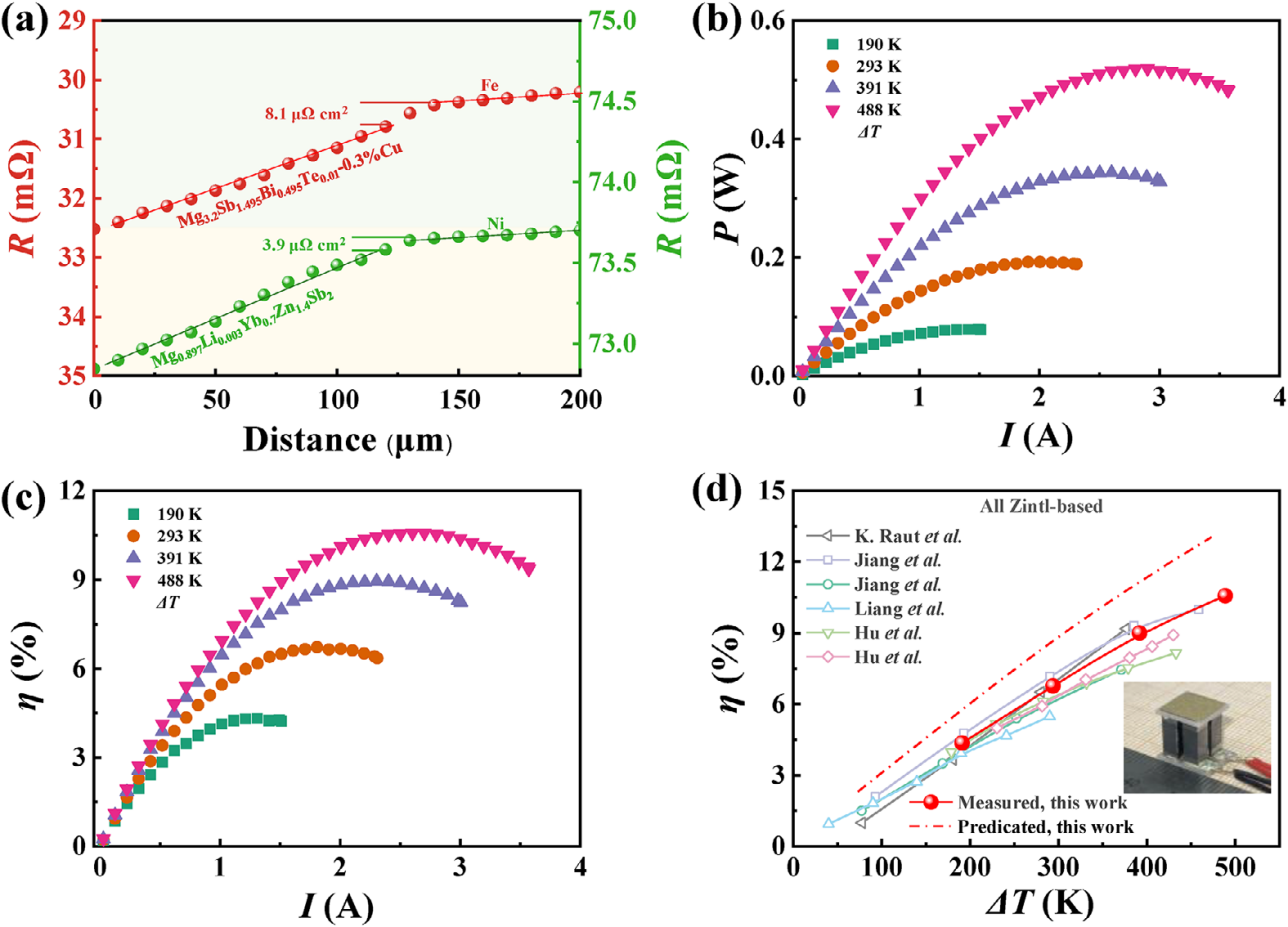
(a) Contact resistivities for the n‐type/Fe junction and p‐type/Ni junction. Current (*I*) dependent (b) output power and (c) conversion efficiency. (d) Comparison of the measured conversion efficiency among all Zintl‐based modules as a function of temperature gradient. The dashed line represents the simulated values.

Using the high‐performance n‐type Mg_3.2_Bi_0.495_Sb_1.495_Te_0.01_‐0.3% Cu material developed in this work, we assembled a two‐pair TE module. Mg_0.897_Li_0.003_Zn_1.4_Yb_0.7_Sb_2_ material was selected as the p‐type leg because it is isostructural to our n‐type material, both crystallizing in the CaAl_2_Si_2_‐type structure. This structural consistency is beneficial for minimizing thermal expansion mismatch and enhancing the mechanical reliability of the module during thermal cycling. Fe and Ni were adopted as the interfacial materials for the n‐type and p‐type legs, respectively. The sandwich‐type architecture was fabricated via a one‐step sintering process to simultaneously consolidate the interfacial layers and TE legs. As shown in Figure 6a, the contact resistivity values at the n‐type/Fe junction and p‐type/Ni junction are as low as 8.1 and 3.9 µΩ cm^2^, respectively. These values are competitive with previously reported values for Mg_3_(Sb, Bi)_2_‐based TE devices, as summarized in Figure S9. The low interfacial resistivity guarantees negligible electrical energy loss across the interfaces as well as high open circuit voltage (*V*_oc_) (Figure S10a) and output power (*P*) (Figure 6b). A high output power of 0.52 W is achieved at a temperature gradient *ΔT* of 488 K. The current (*I*) dependent heat flow (*Q*_c_) and heat‐to‐electricity conversion efficiency (*η*) of the module under a series of *ΔT* are plotted in Figure S10b and Figure 6c. The conversion efficiency displays an approximately linear enhancement with increasing temperature gradient. Benefiting from the module design, thermal radiation and interfacial energy losses are effectively minimized, yielding a high conversion efficiency *η* of 10.6% at a *ΔT* of 488 K. This efficiency surpasses most reported high‐performance all‐Zintl TE modules [54, 55, 56, 57, 58, 59], underscoring the strong potential of this system for waste heat recovery applications.

## 3 Conclusion

In summary, we experimentally distinguish the roles of tantalum‐tube encapsulated melting, Cu addition, and Sb/Bi regulation in Mg_3_(Sb, Bi)_2_. Encapsulated melting alone increases the average grain size from 0.9 to 23.6 µm, suppressing grain‐boundary carrier scattering and yielding single‐crystal‐like carrier transport in a polycrystalline system. Cu addition further increases the grain size only slightly to 25.5 µm. APT reveals Mg‐rich grain boundaries in the Cu‐containing sample, while SEM‐EDS and STEM‐EDS identify localized Mg_2_Cu nanoprecipitates at grain‐boundary triple junctions. These results indicate that Cu primarily contributes to grain‐boundary modification and nanoprecipitate formation rather than grain coarsening. The Mg_2_Cu nanoprecipitates, together with Sb/Bi alloy disorder, effectively scatter phonons and reduce *κ*_L_. Further regulation of the Sb/Bi ratio balances carrier mobility, band gap, bipolar excitation, and alloy scattering. Consequently, a peak *zT* of 1.9 at 750 K and a two‐pair all‐Zintl module efficiency of 10.6% at *ΔT* = 488 K are achieved in the Mg_3.2_Bi_0.495_Sb_1.495_Te_0.01_‐0.3% Cu sample. These findings provide in‐depth insights into the application development of Mg_3_(Sb, Bi)_2_‐based materials in the low‐to‐medium temperature region.

## 4 Experimental Section

### 4.1 Sample Preparation and Characterization

Large grain polycrystalline Mg_3.2_Sb_1.995‐_ *_x_* Bi*_x‐_*_0.005_Te_0.01_‐0.3% Cu (0.5 ≤ *x* ≤ 1.6) samples were synthesized by a tantalum‐tube encapsulated melting technique. High purity raw elements Mg (pellets, 99.95%, Aladdin), Bi (shot, 99.999%, Aladdin), Sb (shot, 99.999%, Alfa Aesar), Te (shot, 99.999%, Alfa Aesar), and Cu (shot, 99.999%, Alfa Aesar) were weighed out in stoichiometric proportions, loaded and sealed into a tantalum tube under an argon atmosphere. Subsequently, the tantalum tube was vacuum‐sealed into a quartz tube. These quartz tubes were heated to 1423 K over 11 h, maintained at this temperature for 24 h, and then naturally cooled to room temperature. The obtained ingot was manually ground into large granular chunks of 1–2 mm in a glove box. For the preparation of the Mg_3.2_Bi_1.195_Sb_0.795_Te_0.01_ sample via ball milling, the same starting materials were filled into a stainless‐steel vessel in an argon‐filled glove box, and then ball milled using a vibratory ball mill (MSK‐SFM‐3‐1, Hefei Kejing Materials Technology Co., Ltd) at 1200 rpm for 5 h. Both melted and ball‐milled materials were densified by a spark plasma sintering system (SPS‐222H, Fuji Electronic Industrial Co., Ltd) at 893 K under an axial pressure of 50 MPa for 3 min. Since the melting point of Mg_3_Bi_2_ is lower than that of Mg_3_Sb_2_, the sintering temperatures of Mg_3.2_Sb_1.995‐_ *_x_* Bi*_x‐_*_0.005_Te_0.01_‐0.3% Cu during the SPS process are different (923 K for *x =* 0.5, 0.7; 903 K for *x =* 0.9, 1.0, 1.1; 893 K for *x =* 1.2, 1.3; 873 K for *x =* 1.4, 1.5, 1.6), while the pressure and time remain unchanged. Powder X‐ray diffraction data were collected on a Bruker D8 Advance diffractometer. The chemical compositions of the samples were determined by field emission scanning electron microscopy (FESEM, Tescan Mira3, Czech Republic) equipped with energy dispersive X‐ray spectroscopy (EDS, Oxford, UK). Transmission electron microscopy (TEM) examination was conducted with a JEM ARM200F electron microscope (JEOL) fitted with a spherical aberration corrector (CEOS GmbH) for the objective lens system and the TEM sample was prepared by using focused ion beam (FIB) polishing in a TESCAN GAIA3 FIB‐SEM system. Atom‐probe tomography (APT) analysis was performed using a Cameca Instruments (LEAP 5000 XR). 20 million ions were collected by the APT with a 50 K measurement temperature and a detection rate of 1%. The FIB lift‐out method (Helios, Nanolab 600) was employed to prepare needle‐shaped specimens for APT analysis. The electrical properties (*σ* and *S*) were measured using a commercial ZEM‐3 system (ULVAC Co. Ltd) under a helium atmosphere. Thermal diffusivity (*D*) was measured in an argon atmosphere using a laser flash method (LFA 457, Netzsch Co. Ltd). Density (*ρ*) was measured via the Archimedes principle. Heat capacity (*C*_p_) was estimated using the Dulong–Petit law. Accordingly, the total thermal conductivity (*κ*) was calculated using the formula *κ* = *ρC*_p_ *D*. Uncertainties in the electrical conductivity, Seebeck coefficient, and thermal conductivity were within 5%, 7%, and 5%, respectively. The physical property measurement system (PPMS‐9, Quantum Design) was used to measure the Hall coefficient at 300 K with a magnetic field swept from −3 to 3 T.

### 4.2 TE Module Fabrication and Measurements

The overall size of the two‐couple TE module, consisting of p‐type Ni/Mg_0.897_Zn_1.4_Yb_0.7_Sb_2_Li_0.003_/Ni and n‐type Fe/Mg_3.2_Bi_0.495_Sb_1.495_Te_0.01_‐0.3% Cu/Fe legs, was 10×10×10.5 mm^3^. Sandwich‐structured p‐ and n‐type TE legs were fabricated by a one‐step SPS technique. Both of them are sintered at 923 K for 3 min under an axial pressure of 50 MPa. The dimensions of the p‐ and n‐type TE legs were 3.5×3.5×8.5 mm^3^ and 3×3×8.5 mm^3^, respectively. The hot side was bonded to the copper‐plated aluminum nitride ceramic substrate using a silver nanocomposite paste at 573 K, yielding mechanically reliable and electrically ohmic contacts. The cold side of the TE legs was joined with a copper electrode using solder paste. Four copper wires were subsequently soldered to the cold side for current and voltage measurements. The conversion efficiency of the two‐couple TE module was evaluated using a home‐built measurement system capable of maintaining precise temperature control under fluctuating hot‐side temperatures [53]. All measurements were performed in an argon‐purged chamber under a uniaxial pressing force of 250 N to reduce both electrical and thermal contact resistances. The hot side temperature was regulated by a resistive heater, while the cold side was held at ∼283 K through circulating water.

## Author Contributions

**Jingdan Lei**: writing – original draft, conceptualization, investigation, writing – review & editing, methodology, data curation, validation, formal analysis. visualization. **Kai Xu**: methodology, data curation, validation, formal analysis. **Kunpeng Zhao**: conceptualization, writing – original draft, supervision, writing – review & editing, project administration, methodology, investigation, funding acquisition, formal analysis. **Junzhuo Zhou**: methodology, formal analysis. **Haotian Gao**: methodology, formal analysis. **Yifan Yuan**: methodology, formal analysis. **Tian‐Ran Wei**: investigation, methodology, resources, funding acquisition, project administration. **Min Zhu**: writing – review & editing, methodology, formal analysis. **Qihao Zhang**: formal analysis, writing – review & editing, visualization, supervision. **Franck Gascoin**: writing – review & editing, formal analysis. **Xun Shi**: writing – review & editing, methodology, funding acquisition, supervision, project administration.

## Conflicts of Interest

The authors declare no conflicts of interest.

## Supporting information




**Supporting File**: advs77449‐sup‐0001‐SuppMat.docx.

## Data Availability

The data that support the findings of this study are available from the corresponding author upon reasonable request.
