# Supplementary material for: Synergistic Grain Boundary and Phonon Engineering Enables Ultrahigh Thermoelectric Performance in Mg3(Sb, Bi)2‐Based Materials
Source: Adv Sci (Weinh). 2026 Aug 30:e77449. Online ahead of print. doi: 10.1002/advs.77449 (PMC13526534; doi:10.1002/advs.77449)
Supplement: Supplementary file 1 [file ADVS-9999-e77449-s001.docx]

**Supporting information**

**Synergistic Grain Boundary and Phonon Engineering Enables Ultrahigh Thermoelectric Performance in Mg_3_(Sb, Bi)_2_-based Materials**

*Jingdan Lei^1,2,3,#^, Kai Xu^4,#^, Kunpeng Zhao^2,3*^, Junzhuo Zhou^2,3^, Haotian Gao^2,3^, Yifan Yuan^2,3^, Tian-Ran Wei^2,3^, Min Zhu^5^, Qihao Zhang^4*^, Franck Gascoin^6^, and Xun Shi^2,7*^*

**Callaway model**

To quantitatively evaluate the point-defect scattering arising from Sb/Bi disorder, we employed the Callaway-Klemens alloy scattering model. The ratio of the alloy thermal conductivity *κ*_L,_*_x_* to that of the non-alloyed counterpart *κ*_L,0_ is given by:

$$\frac{\kappa_{\text{L,}\text{x}}}{\kappa_{\text{L,}\text{0}}}=\frac{\arctan(u)}{u},$$

with

$$u^{2}=\frac{\pi\theta_{\text{D}}\Omega}{2\hbar\nu^{2}}\kappa_{\text{L,}\text{0}}\Gamma.$$

Here, $\theta_{\text{D}}$ is the Debye temperature, $\Omega$ is the atomic volume per site, $\nu$ is the average speed of sound, and $\Gamma$ is the point-defect scattering parameter. $\Gamma$ is presented by:

$$\Gamma=\Gamma_{M}+\Gamma_{S}$$

where *Γ*_M_ and *Γ*_S_ are the mass-fluctuation and strain-field fluctuation terms, respectively, given by^[1]^:

$$\Gamma_{\text{M}}=\frac{\sum_{i} c_{i}f_{i}^{\text{Sb}}f_{i}^{\text{Bi}}\left( \frac{M_{i}^{\text{Sb}}-M_{i}^{\text{Bi}}}{\bar{M}} \right)^{2}}{\sum_{i} c_{i}},$$

$$\Gamma_{\text{S}}=\frac{\sum_{i} c_{i}f_{i}^{\text{Sb}}f_{i}^{\text{Bi}}\varepsilon\left( \frac{r_{i}^{\text{Sb}}-r_{i}^{\text{Bi}}}{\bar{r}} \right)^{2}}{\sum_{i} c_{i}},$$

where *M*_i_, *r*_i_ are the relative atomic mass and ionic radius of the atom occupying site *i*, respectively. $\bar{M}$ and $\bar{r}$ are the corresponding average values for the alloy, and $\varepsilon$ is the strain-field scaling parameter.

**Figure S1.** Backscattering scanning electron (BSE) images and corresponding energy dispersive spectrometry (EDS) mapping: **(a)** Mg_3.2_Bi_1.195_Sb_0.795_Te_0.01_ synthesized by ball milling, **(b)** Mg_3.2_Bi_1.195_Sb_0.795_Te_0.01_ synthesized by melting, and melted Mg_3.2_Bi_1.395_Sb_0.595_Te_0.01_-0.3% Cu.

**Figure S2.** Temperature dependent thermal conductivity *κ* for ball-milled Mg_3.2_Bi_1.195_Sb_0.795_Te_0.01_, melted Mg_3.2_Bi_1.195_Sb_0.795_Te_0.01_, and melted Mg_3.2_Bi_1.195_Sb_0.795_Te_0.01_-0.3% Cu.

**Figure S3.** **(a)** Room-temperature powder XRD patterns and **(b)** lattice parameters calculated by structural Rietveld refinement. The data from literatures are included for comparison^[2]^.

**Figure S4**. The Rietveld refinement results according to XRD patterns of Mg_3.2_Sb_2-_*_x_*Bi*_x_* (0.5 ≤ *x* ≤ 1.6) samples.

**Figure S5.** Calculated disorder parameter *Γ*, consisting of contribution from mass fluctuations *Γ*_M_ and strain field fluctuations *Γ*_S_.

**Figure S6.** Thermoelectric properties of Cu-free and Cu-containing Mg_3.2_Sb_1.995-_*_x_*Bi*_x_*_-0.005_ (*x =* 0.5 and 1.2) samples. **(a)** Seebeck coefficient *S*, **(b)** electrical conductivity *σ*, **(c)** thermal conductivity *κ*, and **(d)** TE figure of merit *zT*.

**Figure S7.** Repeatable testing of **(a)** Seebeck coefficient *S*, **(b)** electrical conductivity *σ*, **(c)** thermal conductivity *κ*, and **(d)** TE figure of merit *zT* for Mg_3.2_Sb_1.995-_*_x_*Bi*_x_*_-0.005_ (*x =* 0.5 and 1.2) samples.

**Figure S8**. SEM images and corresponding EDS elemental mappings of the Mg_3.2_Sb_1.495_Bi_0.495_Te_0.01_-0.3% Cu sample **(a)** before and **(b)** after the thermal cycling tests.

**Figure S9.** Comparison of contact resistivity at n-type Mg_3_(Sb,Bi)_2_ interfaces ^[3]^ ^[4]^ ^[5]^.

**Figure S10.** Current (*I*) dependence of **(a)** open circuit voltage (*V*_oc_) and **(b)** heat flow (*Q*_c_) under different temperature gradient.

**Table S1** The full width at half maximum (FWHM) of the typical patterns using Williamson-Hall analysis for the three samples.

|  | Ball milling (°) | Melting (°) | Melting + Cu (°) |
| --- | --- | --- | --- |
| 22.1° (100) | 0.46 | 0.34 | 0.26 |
| 24.2° (002) | 0.4 | 0.26 | 0.2 |
| 25.3° (011) | 0.48 | 0.34 | 0.24 |
| 33.1° (012) | 0.72 | 0.37 | 0.31 |
| 39.0° (110) | 0.73 | 0.32 | 0.30 |
| 43.3° (103) | 0.7 | 0.36 | 0.32 |

**References**

[1]	a)P. G. Klemens, *Proceedings of the Physical Society. Section A* **1955**, *68*, 1113; b)B. Abeles, *Phys. Rev.* **1963**, *131*, 1906-1911; c)J. Yang, G. P. Meisner, L. Chen, *Appl. Phys. Lett.* **2004**, *85*, 1140-1142.

[2]	a)X. Shi, X. Zhang, A. Ganose, J. Park, C. Sun, Z. Chen, S. Lin, W. Li, A. Jain, Y. Pei, *Mater. Today Phys.* **2021**, *18*, 100362; b)Z. Han, Z. Gui, Y. B. Zhu, P. Qin, B.-P. Zhang, W. Zhang, L. Huang, W. Liu, *Research* **2020**, *2020*, 12, 1672051; c)S. W. Song, J. Mao, M. Bordelon, R. He, Y. M. Wang, J. Shuai, J. Y. Sun, X. B. Lei, Z. S. Ren, S. Chen, S. Wilson, K. Nielsch, Q. Y. Zhang, Z. F. Ren, *Mater. Today Phys.* **2019**, *8* 25-33.

[3]	a)M. F. Aamir, R. Chetty, J. Babu, T. Mori, *J. Mater. Chem. C* **2025**, *13*, 10567-10575; b)L. Yin, C. Chen, F. Zhang, X. Li, F. Bai, Z. Zhang, X. Wang, J. Mao, F. Cao, X. Chen, J. Sui, X. Liu, Q. Zhang, *Acta Mater.* **2020**, *198*, 25-34.

[4]	a)Z. Bu, X. Zhang, Y. Hu, Z. Chen, S. Lin, W. Li, Y. Pei, *Energy Environ. Sci.* **2021**, *14*, 6506-6513; b)P. Ying, L. Wilkens, H. Reith, N. P. Rodriguez, X. Hong, Q. Lu, C. Hess, K. Nielsch, R. He, *Energy Environ. Sci.* **2022**, *15*, 2557-2566; c)X. Wu, Z. Han, Y. Zhu, B. Deng, K. Zhu, C. Liu, F. Jiang, W. Liu, *Acta Mater.* **2022**, *226*, 117616.

[5]	a)Y. Fu, Q. Zhang, Z. Hu, M. Jiang, A. Huang, X. Ai, S. Wan, H. Reith, L. Wang, K. Nielsch, W. Jiang, *Energy Environ. Sci.* **2022**, *15*, 3265-3274; b)R. Chetty, J. Babu, T. Mori, *ACS Appl. Energy Mater.* **2024**, *7*, 12112-12118; c)Y. Fu, X. Ai, Z. Hu, S. Zhao, X. Lu, J. Huang, A. Huang, L. Wang, Q. Zhang, W. Jiang, *Nat. Commun.* **2024**, *15*, 9355; d)N. Qu, Y. Sun, Z. Liu, L. Xie, Y. Zhu, H. Wu, R. Chai, J. Cheng, F. Guo, Q. Zhang, R. Wang, B. Li, Z. Wei, W. Cai, J. Sui, *Adv. Energy Mater.* **2024**, *14*, 2302818.
